# (*E*,*E*)-1,2-Bis[3-(prop-2-yn-1-yl­oxy)benzyl­idene]hydrazine

**DOI:** 10.1107/S1600536812025524

**Published:** 2012-06-13

**Authors:** Wisam Naji Atiyah Al-Mehana, Rosiyah Yahya, Faridah Sonsudin, Kong Mun Lo

**Affiliations:** a Department of Chemistry, University of Malaya, 50603 Kuala Lumpur, Malaysia; b Centre for Foundation Studies in Science, University of Malaya, 50603 Kuala Lumpur, Malaysia

## Abstract

The mol­ecule of the title compound, C_20_H_16_N_2_O_2_, is centrosymmetric, the inversion center being located at the mid-point of the central azine bond. The conformation around the C=N bond is *E*. The whole mol­ecule (except for the H atoms) is essentially planar, with an r.m.s. deviation of 0.07 Å. In the crystal, mol­ecules are linked head-to-tail by pairs of C—H⋯O hydrogen bonds, forming inversion dimers, and resulting in the formation of chains propagating along [011].

## Related literature

For biological properties and practical appplications of diacetyl­ene compounds, see: Zloh *et al.* (2007 ▶); Buckley & Neumeister (1992 ▶). For the structure of (*E,E*)-1,2-bis­[3-(prop-2-yn-1-yl­oxy)benzyl­indene]­hydrazine see: Al-Mehana *et al.* (2011 ▶).
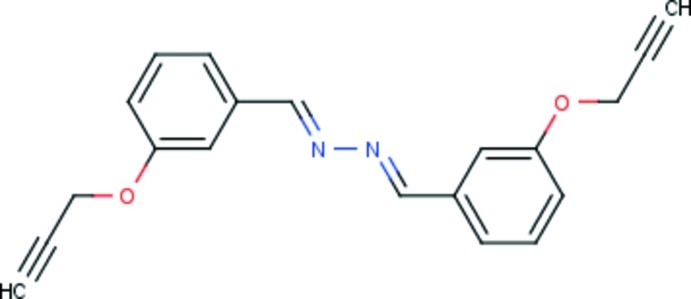


## Experimental

### 

#### Crystal data

C_20_H_16_N_2_O_2_
*M* *_r_* = 316.35
Triclinic, 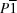

*a* = 4.5700 (3) Å
*b* = 9.4947 (7) Å
*c* = 9.8920 (8) Å
α = 67.986 (7)°
β = 77.487 (6)°
γ = 84.132 (6)°
*V* = 388.37 (5) Å^3^
*Z* = 1
Mo *K*α radiation
μ = 0.09 mm^−1^
*T* = 100 K
0.1 × 0.08 × 0.08 mm

#### Data collection

Agilent SuperNova Dual (Cu) Atlas diffractometer
Absorption correction: multi-scan (*CrysAlis PRO*; Agilent, 2012 ▶) *T*_min_ = 0.440, *T*_max_ = 1.000
2956 measured reflections
1710 independent reflections
1508 reflections with *I* > 2σ(*I*)
*R*_int_ = 0.027

#### Refinement

*R*[*F* ^2^ > 2σ(*F* ^2^)] = 0.041
*wR*(*F* ^2^) = 0.122
*S* = 1.02
1710 reflections
109 parameters
H-atom parameters constrained
Δρ_max_ = 0.23 e Å^−3^
Δρ_min_ = −0.29 e Å^−3^

### 

Data collection: *CrysAlis PRO* (Agilent, 2012 ▶); cell refinement: *CrysAlis PRO*; data reduction: *CrysAlis PRO*; program(s) used to solve structure: *SHELXS97* (Sheldrick, 2008 ▶); program(s) used to refine structure: *SHELXL97* (Sheldrick, 2008 ▶); molecular graphics: *X-SEED* (Barbour, 2001 ▶); software used to prepare material for publication: *pubCIF* (Westrip, 2010 ▶).

## Supplementary Material

Crystal structure: contains datablock(s) I, global. DOI: 10.1107/S1600536812025524/su2430sup1.cif

Structure factors: contains datablock(s) I. DOI: 10.1107/S1600536812025524/su2430Isup2.hkl

Supplementary material file. DOI: 10.1107/S1600536812025524/su2430Isup3.cml

Additional supplementary materials:  crystallographic information; 3D view; checkCIF report

## Figures and Tables

**Table 1:** Hydrogen-bond geometry (Å, °)

*D*—H⋯*A*	*D*—H	H⋯*A*	*D*⋯*A*	*D*—H⋯*A*
C5—H5⋯O1^i^	0.93	2.52	3.4467 (16)	177

Symmetry code: (i) 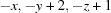.

**Fig. 1.**
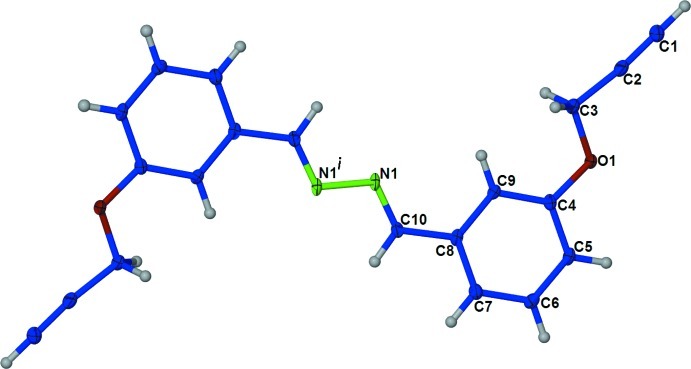
The molecular structure of the title molecule, with the atom numbering. The displacement ellipsoids are drawn at the 50% probability level [symmetry code: (i) -x+2, -y+1, -z+2].

**Fig. 2.**
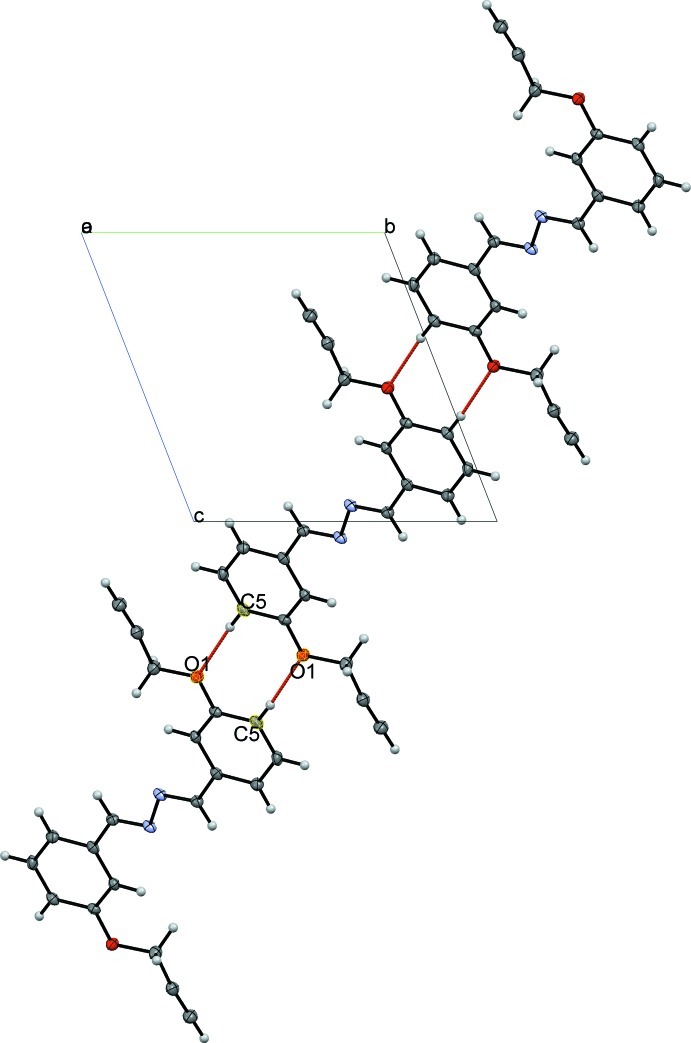
A partial view along the a axis of the crystal packing of the title compound, illustrating the formation of the intermolecular chain along [011]. The C—H···O interactions are shown as red lines; see Table 1 for details.

## supplementary crystallographic information

### Comment

Diacetylene compounds are known to have antitumour and antimicrobial activities (Zloh *et al.*, 2007). In addition to their biological activities, diacetylenes are also used as coating and surface treatment agents and as inner cladding material for silica fibre-optic cores (Buckley & Neumeister, 1992). A recent study detailed the crystal structure of (*E,E*)-1,2-Bis[4-(prop-2-yn-1-yloxy)benzylindene]hydrazine (Al-Mehana *et al.*, 2011). Herein we report on the synthesis and crystal structure of the 3-substituted isomer of this diacetylene compound.

Like the above mentioned compound, the title compound, Fig. 1, is also centrosymmetric around the central azine bond [N1—N1^i^ = 1.415 (1) Å; Symmetry code: (i) -x+2, -y+1, -z+2], with an *E* conformation about the N1═C10 bond [1.2810 (18) Å].

The title compound differs from the 4-substituted isomer (Al-Mehana *et al.*, 2011) as it adopts a different type of C—H···O interaction in its crystal packing. Here, one of the aromatic H atoms is a hydrogen bond donor to the adjacent phenoxy O atom resulting in the formation of an infinite polymeric chain propagating along [011] (Table 1 and Fig. 2).

### Experimental

3,3'-(*E*, *E*)-hydrazine-1,2-diylidene bis(methan-1-yl-1-ylidene)diphenol (*L*_1_) was prepared by stirring 3-hydroxybenzaldehyde (3 g, 24.5 mmol), hydrazine sulfate (1.65 g, 12.6 mmol) and 1.5 ml of concentrated ammonium solution in a mixture of ethanol and water (20 ml) for 3 h. The product was obtained as a yellow crystalline solid, m.p. 487 - 488 K. A mixture of the diphenol, *L*_1_ (2 g, 8.3 mmol) and anhydrous potassium carbonate (1.84 g, 8.6 mmol) in 20 ml of dry acetone was stirred for 30 minutes. Then an excess of propargyl bromide (2.28 g, 19.2 mmol) was added drop wise and the resulting mixture was left under reflux for 48 h. The solvent was then evaporated under reduced pressure. The product was extracted with 100 ml of diethyl ether. The organic layer was washed with brine and dried with MgSO_4_. A yellow amorphous solid was obtained upon slow evaporation of the ethereal solution and was recrystallized with a 1:1 ethyl acetate-methanol mixture, to yield pure yellow block-like crystals of the title compound [M.p. 401 - 403 K].

### Refinement

Hydrogen atoms were included in calculated positions and treated as riding atoms: C–H 0.93 Å (CH) and 0.97 Å (CH_2_) with *U*_iso_(H) = 1.2*U*_eq_(C).

### Crystal data

**Table:**

C_20_H_16_N_2_O_2_	*Z* = 1
*M**_r_* = 316.35	*F*(000) = 166
Triclinic, *P*1	*D*_x_ = 1.353 Mg m^−^^3^
Hall symbol: -P 1	Melting point = 401–403 K
*a* = 4.5700 (3) Å	Mo *K*α radiation, λ = 0.71073 Å
*b* = 9.4947 (7) Å	Cell parameters from 1636 reflections
*c* = 9.8920 (8) Å	θ = 2.3–29.1°
α = 67.986 (7)°	µ = 0.09 mm^−^^1^
β = 77.487 (6)°	*T* = 100 K
γ = 84.132 (6)°	Block, colourless
*V* = 388.37 (5) Å^3^	0.1 × 0.08 × 0.08 mm

### Data collection

**Table:**

Agilent SuperNova Dual (Cu at zero) Atlas diffractometer	1710 independent reflections
Radiation source: SuperNova (Mo) X-ray Source	1508 reflections with *I* > 2σ(*I*)
Mirror monochromator	*R*_int_ = 0.027
ω scans	θ_max_ = 27.5°, θ_min_ = 2.3°
Absorption correction: multi-scan (*CrysAlis PRO*; Agilent, 2012)	*h* = −5→4
*T*_min_ = 0.440, *T*_max_ = 1.000	*k* = −12→12
2956 measured reflections	*l* = −12→12

### Refinement

**Table:**

Refinement on *F*^2^	Primary atom site location: structure-invariant direct methods
Least-squares matrix: full	Secondary atom site location: difference Fourier map
*R*[*F*^2^ > 2σ(*F*^2^)] = 0.041	Hydrogen site location: inferred from neighbouring sites
*wR*(*F*^2^) = 0.122	H-atom parameters constrained
*S* = 1.02	*w* = 1/[σ^2^(*F*_o_^2^) + (0.0756*P*)^2^ + 0.0718*P*] where *P* = (*F*_o_^2^ + 2*F*_c_^2^)/3
1710 reflections	(Δ/σ)_max_ < 0.001
109 parameters	Δρ_max_ = 0.23 e Å^−^^3^
0 restraints	Δρ_min_ = −0.29 e Å^−^^3^

### Special details

**Table:**

Geometry. Bond distances, angles etc. have been calculated using the rounded fractional coordinates. All su's are estimated from the variances of the (full) variance-covariance matrix. The cell esds are taken into account in the estimation of distances, angles and torsion angles
Refinement. Refinement of *F*^2^ against ALL reflections. The weighted *R*-factor *wR* and goodness of fit *S* are based on *F*^2^, conventional *R*-factors *R* are based on *F*, with *F* set to zero for negative *F*^2^. The threshold expression of *F*^2^ > σ(*F*^2^) is used only for calculating *R*-factors(gt) *etc*. and is not relevant to the choice of reflections for refinement. *R*-factors based on *F*^2^ are statistically about twice as large as those based on *F*, and *R*- factors based on ALL data will be even larger.

### Fractional atomic coordinates and isotropic or equivalent isotropic displacement parameters (Å^2^)

**Table:**

	*x*	*y*	*z*	*U*_iso_*/*U*_eq_	
O1	0.26162 (18)	0.81143 (9)	0.53750 (8)	0.0186 (3)	
N1	0.9141 (2)	0.53809 (10)	0.94457 (10)	0.0154 (3)	
C1	0.1926 (3)	0.64779 (13)	0.28726 (13)	0.0223 (3)	
C2	0.2727 (3)	0.67068 (13)	0.38360 (13)	0.0201 (3)	
C3	0.3877 (3)	0.68058 (13)	0.50670 (13)	0.0202 (3)	
C4	0.3322 (2)	0.82703 (12)	0.65948 (12)	0.0150 (3)	
C5	0.1900 (3)	0.94943 (13)	0.69477 (13)	0.0173 (3)	
C6	0.2352 (3)	0.97068 (12)	0.81942 (13)	0.0186 (3)	
C7	0.4213 (3)	0.87082 (13)	0.90930 (13)	0.0176 (3)	
C8	0.5677 (2)	0.75107 (12)	0.87209 (12)	0.0151 (3)	
C9	0.5252 (2)	0.72894 (12)	0.74544 (12)	0.0148 (3)	
C10	0.7639 (2)	0.65050 (12)	0.96900 (12)	0.0150 (3)	
H1	0.13000	0.62990	0.21190	0.0270*	
H3A	0.33630	0.59000	0.59440	0.0240*	
H3B	0.60440	0.68700	0.48080	0.0240*	
H5	0.06580	1.01620	0.63490	0.0210*	
H6	0.14090	1.05210	0.84350	0.0220*	
H7	0.44750	0.88440	0.99420	0.0210*	
H9	0.62480	0.64970	0.71920	0.0180*	
H10	0.78030	0.66930	1.05280	0.0180*	

### Atomic displacement parameters (Å^2^)

**Table:**

	*U*^11^	*U*^22^	*U*^33^	*U*^12^	*U*^13^	*U*^23^
O1	0.0223 (5)	0.0187 (4)	0.0173 (4)	0.0091 (3)	−0.0109 (3)	−0.0080 (3)
N1	0.0141 (5)	0.0152 (5)	0.0162 (5)	0.0017 (4)	−0.0086 (4)	−0.0022 (4)
C1	0.0254 (6)	0.0225 (6)	0.0209 (6)	0.0061 (5)	−0.0105 (5)	−0.0083 (5)
C2	0.0206 (6)	0.0183 (6)	0.0204 (6)	0.0069 (4)	−0.0071 (4)	−0.0061 (5)
C3	0.0218 (6)	0.0209 (6)	0.0208 (6)	0.0085 (5)	−0.0101 (5)	−0.0098 (5)
C4	0.0146 (5)	0.0156 (5)	0.0135 (5)	−0.0005 (4)	−0.0045 (4)	−0.0027 (4)
C5	0.0166 (6)	0.0146 (5)	0.0184 (6)	0.0037 (4)	−0.0076 (4)	−0.0021 (4)
C6	0.0205 (6)	0.0132 (5)	0.0225 (6)	0.0049 (4)	−0.0069 (5)	−0.0069 (4)
C7	0.0194 (6)	0.0170 (5)	0.0184 (5)	0.0014 (4)	−0.0082 (4)	−0.0065 (4)
C8	0.0126 (5)	0.0136 (5)	0.0175 (5)	−0.0001 (4)	−0.0050 (4)	−0.0028 (4)
C9	0.0142 (5)	0.0128 (5)	0.0166 (5)	0.0026 (4)	−0.0044 (4)	−0.0044 (4)
C10	0.0150 (5)	0.0152 (5)	0.0160 (5)	0.0000 (4)	−0.0067 (4)	−0.0050 (4)

### Geometric parameters (Å, º)

**Table:**

O1—C3	1.4247 (16)	C8—C9	1.4009 (16)
O1—C4	1.3763 (13)	C8—C10	1.4649 (15)
N1—C10	1.2808 (15)	C1—H1	0.9300
N1—N1^i^	1.4158 (13)	C3—H3A	0.9700
C1—C2	1.1883 (18)	C3—H3B	0.9700
C2—C3	1.4632 (18)	C5—H5	0.9300
C4—C5	1.3951 (18)	C6—H6	0.9300
C4—C9	1.3899 (15)	C7—H7	0.9300
C5—C6	1.3817 (17)	C9—H9	0.9300
C6—C7	1.3941 (18)	C10—H10	0.9300
C7—C8	1.3883 (18)		
			
C3—O1—C4	115.61 (9)	O1—C3—H3A	110.00
N1^i^—N1—C10	111.29 (9)	O1—C3—H3B	110.00
C1—C2—C3	173.13 (14)	C2—C3—H3A	110.00
O1—C3—C2	109.82 (11)	C2—C3—H3B	110.00
O1—C4—C5	115.01 (10)	H3A—C3—H3B	108.00
O1—C4—C9	124.05 (11)	C4—C5—H5	120.00
C5—C4—C9	120.93 (11)	C6—C5—H5	120.00
C4—C5—C6	119.44 (12)	C5—C6—H6	120.00
C5—C6—C7	120.38 (12)	C7—C6—H6	120.00
C6—C7—C8	120.04 (11)	C6—C7—H7	120.00
C7—C8—C9	120.09 (10)	C8—C7—H7	120.00
C7—C8—C10	117.97 (10)	C4—C9—H9	120.00
C9—C8—C10	121.94 (10)	C8—C9—H9	120.00
C4—C9—C8	119.08 (11)	N1—C10—H10	118.00
N1—C10—C8	123.54 (10)	C8—C10—H10	118.00
C2—C1—H1	180.00		
			
C3—O1—C4—C9	3.31 (15)	C4—C5—C6—C7	0.0 (2)
C4—O1—C3—C2	174.74 (10)	C5—C6—C7—C8	1.4 (2)
C3—O1—C4—C5	−175.53 (10)	C6—C7—C8—C10	179.20 (11)
C10—N1—N1^i^—C10^i^	179.98 (10)	C6—C7—C8—C9	−0.97 (18)
N1^i^—N1—C10—C8	179.47 (9)	C7—C8—C9—C4	−0.85 (16)
O1—C4—C5—C6	177.00 (11)	C7—C8—C10—N1	−178.79 (11)
C9—C4—C5—C6	−1.88 (18)	C9—C8—C10—N1	1.38 (17)
C5—C4—C9—C8	2.29 (16)	C10—C8—C9—C4	178.97 (10)
O1—C4—C9—C8	−176.49 (10)		

Symmetry code: (i) −*x*+2, −*y*+1, −*z*+2.

### Hydrogen-bond geometry (Å, º)

**Table:**

*D*—H···*A*	*D*—H	H···*A*	*D*···*A*	*D*—H···*A*
C5—H5···O1^ii^	0.93	2.52	3.4467 (16)	177

Symmetry code: (ii) −*x*, −*y*+2, −*z*+1.
